# History and Perspectives of Nuclear Medicine in Thailand

**Published:** 2014

**Authors:** Sombut Boonyaprapa

**Affiliations:** Department of Radiology, Chiang Mai University, Chiang Mai, Thailand

In 1955, the first nuclear medicine division was established in Thailand by Professor Romsai Suwannik at the Department of Radiology of Siriraj Hospital, Mahidol University in Bangkok. Four years later in 1959, the second nuclear medicine division was established at the Department of Radiology of Chulalongkorn Hospital in Bangkok, and the third division was founded at Rajavithi Hospital in Bangkok in 1961. In 1965, ten years after the establishment of the first nuclear medicine division in the country, Professor Dusadee Prabhasavat and Professor Sanan Simarak established the fourth nuclear medicine division at Chiang Mai University, which is the first university located outside Bangkok.

During the early years of nuclear medicine in Thailand (1955-1965), Professor Romsri Suwannik at Siriraj Hospital applied clinical nuclear medicine by using colloidal gold (^198^Au) for palliative treatment of ascites, caused by ovarian cancer. *In vivo* radionuclide non-imaging studies mostly focused on ^131^I thyroid uptake, extracellular fluid volume (^82^Br- distribution space), and exchangeable ^24^Na. ^42^K was studied in cholera patients, and ^32^P was used for radionuclide therapy in malignant pleural effusion and some blood diseases, e.g., leukemia and polycythemia vera. ^32^P was also used for differential diagnosis of benign and malignant pleural effusions.

Since 1965, many radionuclide non-imaging studies have been performed including blood volume studies, ^51^Cr red blood cell (RBC) survival and ferrokinetic studies with ^59^Fe, ^51^Cr platelet survival and kinetic studies with ^125^I-labeled fibrinogen, and ^55^Fe and ^59^Fe absorption studies. Evaluations of renal tubular function using ^131^I-hippuran and renal glomerular function using ^99m^Tc-DTPA are also included in routine clinical services.

For radionuclide imaging studies, ^113m^In-generator and ^198^Au were used in early years before ^99m^Tc generators became available. In 1969, the first radioimmunoassay laboratory was established for clinical services. Also, since 1983, neonatal screening for congenital hypothyroidism has been performed using nuclear medicine techniques in Thailand.

Professor Romsai Suwannik made tremendous efforts for the progress of nuclear medicine in Thailand. One of his well-known studies in nuclear medicine was about thyroid disease in Thailand (Figure 1). He introduced the addition of iodine into drinking water for people living in the Northern and Northeastern parts of the country, which are the endemic areas of goiter in Thailand. He is also known as the father of nuclear medicine in Thailand, and was awarded the medal of Chevalier de l’Ordre du Mérite (Figure 2).

**Figure 1:**
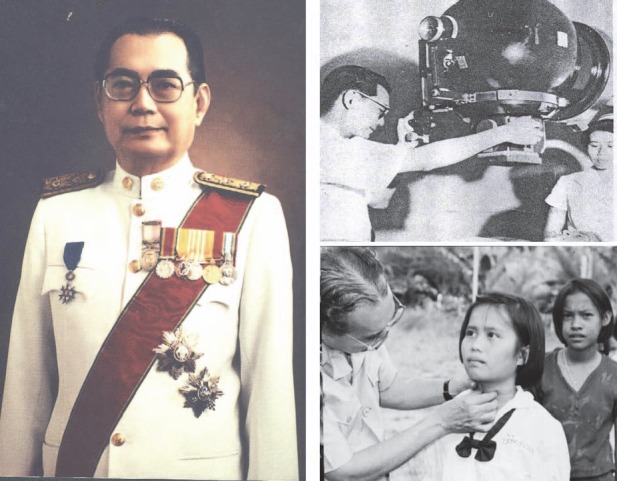
Professor Romsai Suwannik, father of nuclear medicine of Thailand and his works

**Figure 2:**
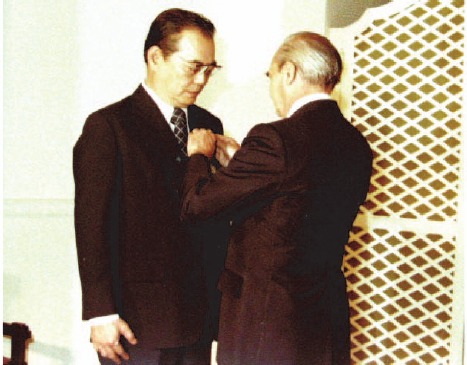
Professor Romsai Suwannik, receiving the medal of Chevalier de l’Ordre du Mérite

Nuclear Medicine Society of Thailand was established in 1982 with 120 members, and Professor Romsai Suwannik was elected as the first president. At present, this organization consists of 227 members, including 78 nuclear medicine physicians.

Many nuclear medicine personnel have made great contributions to the progress of nuclear medicine services in Thailand. Professor Vichai Poshyachinda, who worked during the early years of nuclear medicine in Thailand, developed many clinical nuclear medicine services and performed various studies in Bangkok and other regions of Thailand including Chiang Mai city.

Professor Makumkrong Poshyachinda (Figure 3), the head of Nuclear Medicine Division at Chulalongkorn Hospital in years 1979-1986, and the president of Nuclear Medicine Society of Thailand, was also involved in the development of nuclear medicine services and research in the early years of nuclear medicine. He made great efforts for the progress of nuclear medicine in Thailand.

**Figure 3:**
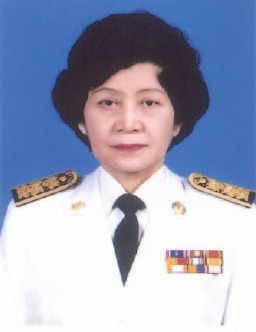
Professor Makumkrong Poshyachinda, the head of nuclear medicine division at the Department of Radiology of Chulalongkorn Hospital, Chulalongkorn University, and the president of Nuclear Medicine Society in Thailand

Many experts in nuclear medicine from other countries have played major roles in the improvement of nuclear medicine services and research in Thailand. These experts are Professor Norman Veall from the U.K, J.D. Pearson from the U.K., Professor Keith Britton from the U.K., Professor Roger P. Ekins from the U.K., Professor Dr. E.H. Belcher from Australia, Ralph Adams from the U.S.A, Professor Donald Germann from the U.S.A, Professor Peter Ell from the U.K., Dr. Marinus Van Kroonenburgh from the Netherlands, Dr. A.K Basu from India, Professor Yasuhito Sasaki from Japan, Professor Shigenobu Nagataki from Japan, Professor Paul Blanget from France, Professor Leif Hallberg from Sweden, and Professor Rosalyn Yalow from the U.S.A.

Most of these nuclear medicine experts are sponsored by the International Atomic Energy Agency (IAEA). They introduced new technologies in clinical services and research and provided training in nuclear medicine.

At present, 25 organizations provide clinical nuclear medicine services in Thailand. Five medical faculties offer three years of nuclear medicine residency training, and eight companies supply radiopharmaceuticals and/or nuclear medicine equipments. One of these faculties is run by the Office of Atoms for Peace in Thailand (OAP of Thailand).

Today, 62 nuclear medicine physicians and 130 non-medical personnel work in Thailand to provide nuclear medicine services. The non-medical personnel include medical physicists, nuclear medicine technologists, radiopharma-cists, radiochemists, and nuclear medicine nurses. Thailand is equipped with eight PET or PET/CT devices for clinical services and research. There are six PET or PET/CT devices in Bangkok, one at Chiang Mai University in the Northern region, and one in Khon Khen University in the Northeastern region of the country.

Most *in vitro* nuclear medicine services include radioimmunoassay of thyroid and other hormones including freeT_4_, freeT_3_, thyroid stimulating hormone (TSH), and thyroxine-binding globulin (TBG) antibodies. Radionuclide ^99m^Tc-labelled compounds, ^131^I, and ^18^F-FDG are routinely used for imaging and clinical nuclear medicine services in Thailand. ^67^Ga, ^68^Ga, ^201^TL, ^111m^In, ^153^Sm, ^131^I, ^188^Re, and ^90^Y are available for clinical diagnosis and radionuclide therapy in some hospitals. All institutes usually perform radionuclide ^99m^Tc-MDP whole body scan, spot planar, and/or single-photon emission computerized tomography (SPECT) imaging for the evaluation of bone metastasis staging and follow-up after treatment in patients with malignant tumours.

Radionuclide ^99m^TcO_4_ thyroid imaging and whole body ^131^I imaging are performed for the diagnosis of thyroid diseases, distant metastasis, and follow-up evaluations after treatment in thyroid carcinoma patients. ^131^I is routinely used for the treatment of patients with hyperthyroid diseases and thyroid carcinoma in Thailand, as shown in Figure 4.

**Figure 4:**
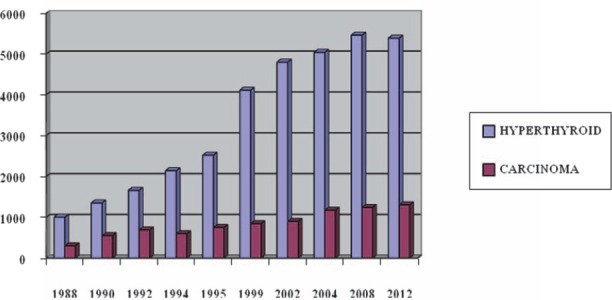
Number of ^131^I therapy for hyperthyroidism and thyroid carcinoma in Thailand

Many institutes perform myocardial perfusion imaging (MPI) with gated SPECT for the diagnosis of myocardial ischemia, myocardial infarction, and evaluation of left ventricular function (Table 1). Multi-gated ^99m^Tc-labeled RBC planar blood pool images or SPECT images are also obtained for the evaluation of left ventricular cardiac function in patients undergoing chemotherapy. The number of all *in vivo* nuclear medicine studies in Thailand is shown in Figure 5.

**Table 1:** The average number of nuclear medicine cardiac imaging studies in year 2011 in Thailand

Nuclear medicine cardiac imaging studies in Thailand in 2011							
Planar Multi-Gated Acquisition (MUGA)	SPECT MUGA	MPI (MIBI)	Gated SPECT (MIBI)	MPI (Tl-201)	Gated SPECT (Tl-201)	Cardiac PET	TOTAL
1000 (22.61%)	11 (0.25%)	1650 (37.31%)	1148 (25.96%)	486 (10.99%)	127 (2.87%)	None (0%)	4422 (100%)

**Figure 5:**
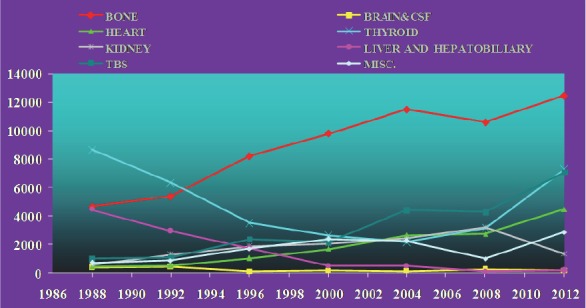
Statistics of clinical nuclear medicine studies in Thailand

Radionuclide ventilation/perfusion (V/Q) pulmonary imaging is usually performed for the diagnosis of pulmonary emboli and evaluation of pulmonary function in patients with pulmonary nodule prior to surgical treatment. However, popularity of radionuclide V/Q pulmonary imaging has decreased since most clinicians prefer pulmonary CT angiography for the diagnosis of pulmonary emboli and pulmonary tumours.

SPECT/CT images are obtained for clinical nuclear medicine services and research. PET/CT images are used for the diagnosis, staging, follow-up, and treatment prediction in patients with malignant tumours, especially non-small cell pulmonary carcinoma and colon and lymphoma carcinomas.

At present, one training course and four educational courses are held in Thailand, which are as follows:

- Thai Board of Nuclear Medicine, three-year training courses for M.D. graduates,
- M.Sc. Program in Medical Physics, two-year training courses after B.Sc. graduate program in radiological technique or scientific physics
- M.Sc. Program in Medical Imaging, two-year educational courses after B.Sc. graduate program in radiological techniques or scientific physics,
- M.Sc. Program in Radiological Sciences, two-year educational courses after B.Sc. graduate program in radiological technology or sciences, and
- B.Sc. Program for Radiological Technologists, four-year educational courses after high school graduation.

There are five nuclear medicine institutes in Thailand which hold nuclear medicine residency training courses. Three of these institutes are located in Bangkok, one in the Northern region, Chiang Mai University, and one in the Northeastern region, Khon Khen University. Overall, 20 residency training positions are available each year at these five nuclear medicine institutes. The residency training course takes three years and includes radiation sciences, clinical sciences, and practice in nuclear medicine under the supervision of nuclear medicine physicians at the nuclear medicine institutes.

After finishing the three-year training, these graduate residents have to take the nuclear medicine national board examination, which is set up every year by Thai national board committee of nuclear medicine. The residents, who pass this board examination, will be certificated to practice as nuclear medicine physicians.

Nuclear medicine research in Thailand, nuclear cardiology studies, and nuclear oncology research are performed in all nuclear medicine institutes, especially at universities, using SPECT, SPECT/CT, and planar imaging systems. Also, research using PET/CT scan is performed at institutes, where PET/CT systems are available.

Academic nuclear medicine meetings for members of nuclear medicine society are held at least twice a year. One is the annual nuclear medicine meeting in Bangkok, as part of the annual academic meeting of Royal College of Radiologists of Thailand, and the other is midyear nuclear medicine academic meeting, which is held by the Nuclear Medicine Society of Thailand. At these two meetings, the findings of nuclear medicine studies of all related institutes are presented. Also, several lectures are presented by nuclear medicine professors from other countries at these meetings each year.

In Thailand, we also have the opportunity to annually organize international academic meetings including IAEA and The Asian Regional Cooperative Council for Nuclear Medicine (ARCCNM) meetings (Figures 6 & 7).

**Figure 6:**
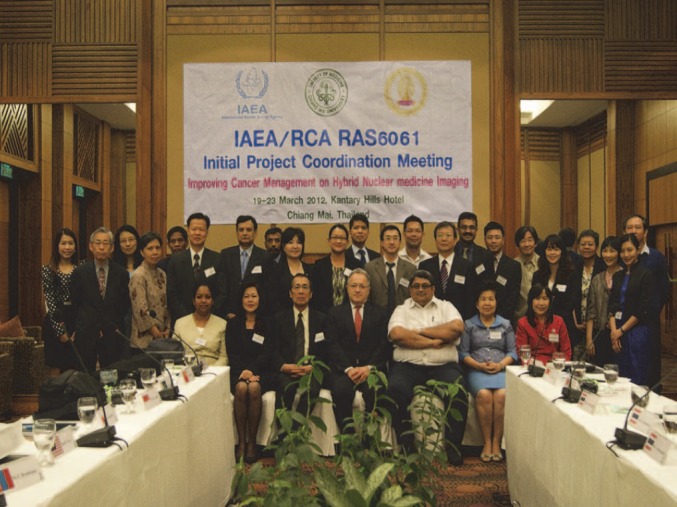
RAS6061/9001/01 IAEA/RCA Initial Project Coordination Meeting in Chiang Mai, Thailand, 19-23 March 2012

**Figure 7:**
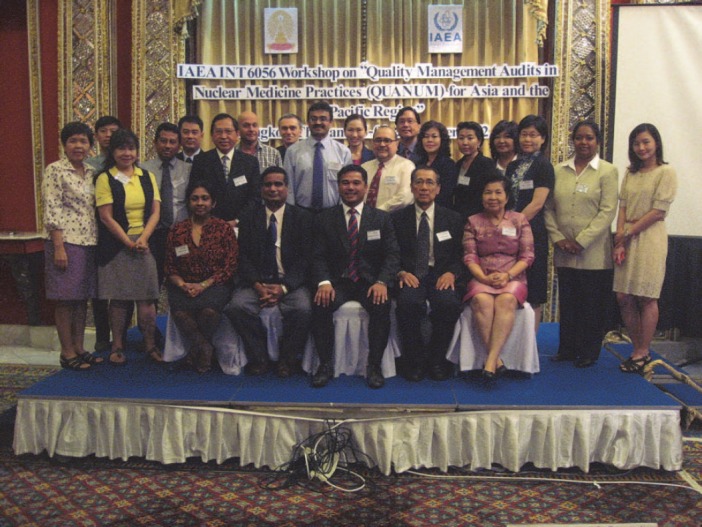
IAEA INT 6056 Workshop on “Quality Management Audits in Nuclear Medicine Practices (QUANUM) for Asia and the Pacific Region” Bangkok, Thailand, 5-9 November, 2012

## Conclusion

Nuclear medicine research and clinical practice in Thailand have greatly improved in recent years and will experience more progress in near future. These achievements contribute to the progress of ARCCNM and Asian countries in this scientific field.

